# Pulmonary Thromboembolism and Myocarditis Resulting from a Large Pacing-Lead-Associated Right Ventricular Thrombus in a Dog with Chronic Cough as Presenting Sign

**DOI:** 10.3390/vetsci11060237

**Published:** 2024-05-24

**Authors:** Viktor Szatmári, Rachel Thomas

**Affiliations:** 1 Department of Clinical Sciences, Faculty of Veterinary Medicine, Utrecht University, 3584 CM Utrecht, The Netherlands; 2 Department Biomolecular Health Sciences, Faculty of Veterinary Medicine, Utrecht University, 3584 CL Utrecht, The Netherlands

**Keywords:** atrio-ventricular block, complication, pacemaker, proteinuria, syncope, ventricular tachycardia

## Abstract

**Simple Summary:**

Dogs with an inappropriately slow heart rate can suffer from decreased endurance and fainting. In addition, they can experience sudden death, and in the long term, they can develop heart failure. Similarly to people, if the slow heart rate cannot be solved with drug therapy, an artificial pacemaker is implanted to increase the heart rate. In most cases, the pacemaker lead is introduced via a large vessel to the heart so that the chest does not have to be opened surgically. Though this procedure is less invasive than suturing the lead on the surface of the heart, it does introduce a foreign body into a great vessel and the heart. The present case report describes a dog, in which the pacemaker lead in the heart caused a large thrombus, which subsequently resulted in pulmonary thromboemboli. The dog presented with a progressively worsening cough and decreased exercise tolerance. Subsequently, lethargy, labored breathing and weakness developed. Because of a suspected bacterial lead-infection, antibiotics were administered, without clinical improvement. Due to worsening clinical signs, the owner elected for euthanasia. Necropsy showed a large thrombus in the right ventricle around the pacing lead and a massive pulmonary thromboembolism. No bacterial infection was apparent.

**Abstract:**

In the present case report, we describe the clinical course and postmortem findings of a 12-year-old Labrador retriever dog with a third-degree atrio-ventricular block that developed a chronic cough, and later dyspnea and weakness as a result of massive pulmonary thromboembolism 3 years after implantation of a transvenous permanent pacemaker. A large soft tissue mass was seen in the right ventricular chamber around the pacing lead with echocardiography. Initially, this was thought to be caused by mural bacterial endocarditis based on hyperthermia, severe leukocytosis and the appearance of runs of ventricular tachycardia, the latter suggesting myocardial damage. While blood culture results were pending, antibiotics were administered without a positive effect. Due to clinical deterioration, the owner elected for euthanasia and a post-mortem examination confirmed a right ventricular thrombus and surrounding myocarditis, without signs of bacterial infection, and a massive pulmonary thromboembolism. We conclude that pulmonary thromboembolism should be considered in dogs with a cough that have an endocardial pacing lead implanted. Serial screening for proteinuria before and after implantation of an endocardial pacing lead would allow timely initiation of prophylactic antiplatelet therapy. Local myocarditis can develop secondary to an intracavitary thrombus, which can subsequently lead to runs of ventricular tachycardia.

## 1. Introduction

Implantation of a permanent pacemaker is the treatment of choice for dogs with pathologic bradycardia that are non-responsive to medical therapy [1,2]. The most common indications are third- and high-grade second-degree atrio-ventricular block, sick sinus syndrome and persistent atrial standstill [1,2]. Transvenous implantation of a ventricular pacing lead is the most commonly used technique as this is less invasive than placing an epicardial lead, which requires either a thoracotomy or laparotomy [3,4].

Pacemaker implantation has numerous possible complications, the most common being lead dislodgement, infection of the pulse generator or the lead, right ventricular wall perforation, and several technical problems with the pacing lead or pulse generator [5,6,7]. Thrombus formation around the intravascular or intracardiac parts of the permanent pacemaker lead has been incidentally reported in larger studies and in a few case reports in dogs [1,5,6,7,8,9], but this complication had not received sufficient attention until a recently published multicenter study that focused on this aspect [10]. According to the authors’ knowledge, there is no detailed report about the clinical course and post-mortem findings of pacemaker-lead-associated thrombosis and subsequent pulmonary thromboembolism in dogs.

## 2. Case Presentation

A 9-year-old intact female Labrador retriever weighing 26 kg was presented to the referring veterinarian because of four syncopal episodes over the course of a day, one week prior to presentation. The referring veterinarian found a bradycardia and referred the dog to an internist. The internist found a heart rate of 44 beats/min. Screening blood examination revealed no abnormalities and an electrocardiogram showed a third-degree atrio-ventricular block. The dog was referred to the authors’ institution for implantation of a permanent artificial pacemaker.

At presentation, the dog was bright, alert and responsive with a respiratory rate of 24 breaths/min and a strong femoral pulse with a rate of 62 beats/min. The rectal temperature was 38.2 °C and the mucous membranes were pink with a capillary refill time of less than a second. Cardiac auscultation revealed a systolic murmur with the point of maximal intensity at the level of the mitral valve, with an intensity of 2 out of 6. Echocardiography showed a mild functional mitral valve regurgitation resulting from a moderate left ventricular eccentric hypertrophy (diastolic left ventricular internal dimension was 51.1 mm, reference range based on body weight 35.6–37.1 mm). No structural cause of the conduction problem was apparent, and a moderate systolic dysfunction of the left ventricle (systolic left ventricular internal dimension was 35.2 mm, reference range based on body weight 22.2–23.5 mm) was seen.

Before general anesthesia for the implantation of the permanent pacemaker, a temporary pacemaker was implanted to guarantee a stable heart rate and rhythm during the procedure. For this, a 4 French introducer was placed in the right saphenous vein with the help of an over-the-needle intravenous catheter and a guidewire. The introducer was secured to the skin with a single suture. Through the introducer, a 4 French temporary pacing lead was inserted and advanced to the right ventricular chamber under fluoroscopic guidance in a manually restrained, non-sedated dog in left lateral recumbency. The temporary pacemaker was switched on, and the rate was set at 80 beats/min. Thereafter, general anesthesia was induced with propofol (170 mg, IV) and an endotracheal tube was inserted. General anesthesia was maintained with inhaled isoflurane with oxygen and fentanyl infusion (10 microgram/kg/h, IV, CRI). Prophylactic antibiotic (a single dose of 520 mg cephazolin, IV) was administered at anesthetic induction. For the implantation of the permanent pacing lead, an approximately 4 cm long skin incision was made parallel and dorsal to the right jugular vein. After ligating the cranial end of the vein with a non-absorbable multifilament suture material (Ethibond 2-0), a 59 cm long permanent bipolar tined pacing electrode with passive fixation (Guidant Selute Picotip) was introduced through an incision of the right jugular vein, and its tip was guided to the right ventricular apex using a curved stylet under fluoroscopic guidance. After removing the stylet and connecting the pacing lead to the pulse generator (Medtronic Adapta ADDR03, Medtronic, Dublin, Ireland), electrocardiography showed 100% capture. There are several measures that can help to make sure that the lead has a good position: (1) the tip of the electrode stays in the same position when the stylet is being removed, (2) feeling resistance when the operator exercises a subtle pull on the lead, (3) the pacemaker programmer attached to the lead shows a clear myocardial injury current, (4) a low (ideally < 1.0 V) stimulation threshold and (5) an appropriate (not too high and not too low) lead impedance. Pacemaker interrogation showed a 0.75 V stimulation threshold with a 0.45 ms impulse and a lead impedance of 549 Ohms. The low stimulation threshold together with this fairly low lead impedance value supports a good contact of the pacing lead with vital myocardium. At the dorsal aspect of the right thoracic wall, caudal to the scapula, a horizontal skin incision was made to create a pocket for the pulse generator under the musculus latissimus dorsi. After disconnecting the pulse generator from the pacing lead, the electrode was tunneled from the skin incision over the jugular vein subcutaneously to this pocket. During this period the temporary pacemaker guaranteed an appropriately high heart rate. The pulse generator was reconnected to the pacing electrode with a screwdriver and the generator was sutured to the underlying soft tissue using non-absorbable multifilament suture material (Ethibond 2-0). The electrode was secured to the jugular vein and to the underlying tissue at the incision site using non-absorbable multifilament suture material with three interrupted sutures (Ethibond 2-0). The temporary pacing electrode was removed under fluoroscopic guidance. Both surgical wounds were closed in two layers using single interrupted sutures, absorbable monofilament for the subcutis (Monocryl 3-0) and non-absorbable monofilament for the skin (Ethilon 3-0). Both surgical wounds were sprayed with a wound spray (Opsite). The pacemaker was set to a rate responsive mode (VVIR 60–105 beats/min) with an impulse amplitude of 2.5 V and 0.45 ms length, and a sensitivity of 2.8 V, using bipolar pacing and bipolar sensing modes. An impulse amplitude of 1.5 V (i.e., twice the pacing threshold) would have been sufficient as the pacing threshold was 0.75 V, but because of the expected increase in threshold in the direct post-implantation period (i.e., so-called acute phase), a higher amplitude was programmed. The reason for choosing 2.5 V was that this value was the highest that still fell under the voltage of the battery of the pulse generator (2.96 V) to spare the battery. The introducer was removed from the right saphenous vein and a pressure bandage was placed on the puncture site for about an hour. The length of surgery was 70 min and the general anesthesia lasted 110 min. Recovery proceeded uneventfully and the dog was discharged from the hospital on the same day. Ten days later the dog returned to the clinic for removal of the skin sutures. The owner reported no more syncopal episodes and no other problems. Physical examination revealed no abnormalities either, a regular femoral pulse with a rate of 80 beats/min was felt. Electrocardiography showed atrio-ventricular dissociation as expected with VVIR mode, 100% paced ventricular rhythm and occasional solitary ventricular premature complexes. Pacemaker interrogation showed no abnormalities: the stimulation threshold was 1.75 V with 0.40 ms and the lead impedance was 1416 Ohm. The increase in threshold and lead impedance is an expected finding in the acute phase after pacemaker implantation due to local inflammation at the myocardium at the pacing site. The stimulation impulse was increased to 3.5 V with 0.52 ms, and the dog was discharged. The reason for increasing the impulse amplitude even above the voltage of the battery was to guarantee the 200% safety margin (2 × 1.75 V = 3.5 V) for myocardial stimulation. If the pulse width had been increased to the maximum (1.0 ms), the pacing amplitude might have been programmed to a somewhat lower value. Because this increase was anticipated to be a temporary measure, and therefore would not drain the battery too much within a few weeks, these parameters were programmed.

**Figure 1:**
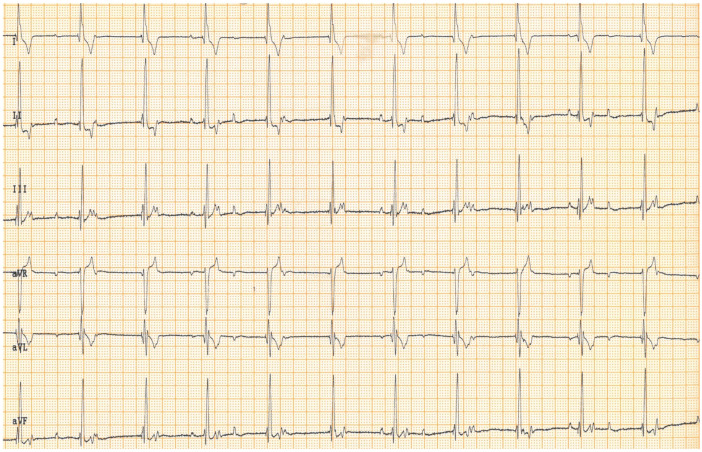
Six-lead surface electrocardiogram of a dog, 2 months after implantation of a permanent pacemaker shows a regular rhythm with atrio-ventricular dissociation, compatible with a 3rd-degree atrio-ventricular block with paced wide ventricular complexes in VVI mode. Paper speed: 25 mm/s, sensitivity: 10 mm/mV.

Routine recheck examination two months after pacemaker implantation revealed no abnormalities in either the history or the physical examination. The pulse rate was 68 beats/min. Electrocardiogram still showed a well-functioning pacemaker in the VVIR setting and a third-degree atrio-ventricular block (Figure 1), and sporadic solitary ventricular premature complexes with left bundle branch block configuration. Pacemaker interrogation showed that the stimulation threshold returned to 0.75 V at 0.52 ms. The pacing impulse was set to 2.0 V and 0.52 ms, and the dog was discharged. The drop in stimulation amplitude is an expected finding two months after the implantation, i.e., after passing the acute phase. The stimulation amplitude was reduced to 2.0 V to increase the battery longevity of the pulse generator.

At a routine check-up six months later, the owner reported no problems, no syncopal episodes and normal exercise tolerance. Physical examination revealed no abnormalities and the femoral pulse rate was 64 beats/min. No echocardiography was performed.

At a routine check-up one year later, the owner reported no problems, normal exercise tolerance and no syncopal episodes. Physical examination revealed no abnormalities and the pulse rate was 66 beats/min. Pacemaker interrogation revealed an unchanged threshold of 0.75 V at 0.52 ms. The lead impedance was 1324 Ohm. At a routine two-year check-up, the owner reported still no problems, no syncopal episodes and normal exercise tolerance. Physical examination revealed no abnormalities and the femoral pulse rate was 76 beats/min. No echocardiography was performed during these yearly rechecks.

Four months after the previous check-up and three years after pacemaker implantation, the owner consulted the referring veterinarian because of daily bouts of coughing occurring 10–15 times a day over the last 5–6 weeks. The dog did not seem ill but endurance was decreased; the dog started to tire and pant after about 30 min of walk, while before the cough started, it was able to walk for more than an hour with ease. The veterinarian found an increased rectal temperature (39.7 °C), and prescribed a course of doxycycline for 10 days. At a check-up two weeks later, the owner reported no improvement in the cough, while the general condition of the dog remained unchanged. The only remarkable finding of the referring veterinarian was periods of tachycardia (rate was not documented) with a basal heart rate of 66 beats/min. A course of oral amoxicillin with clavulanic acid was prescribed and the dog was referred back to the authors’ institution.

**Figure 2:**
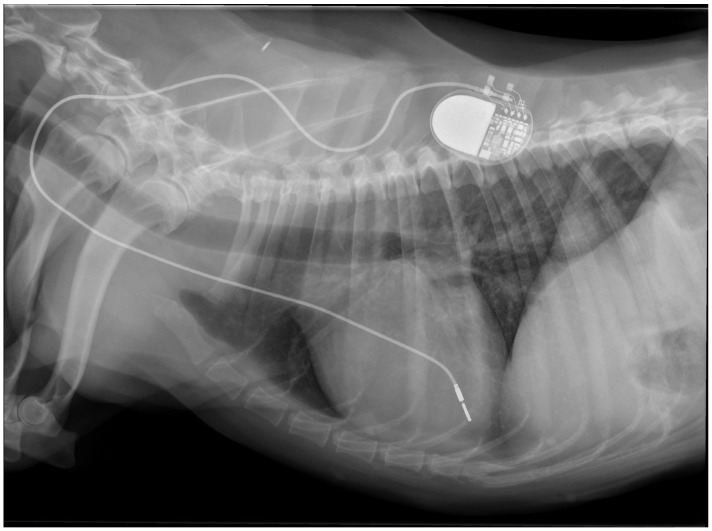

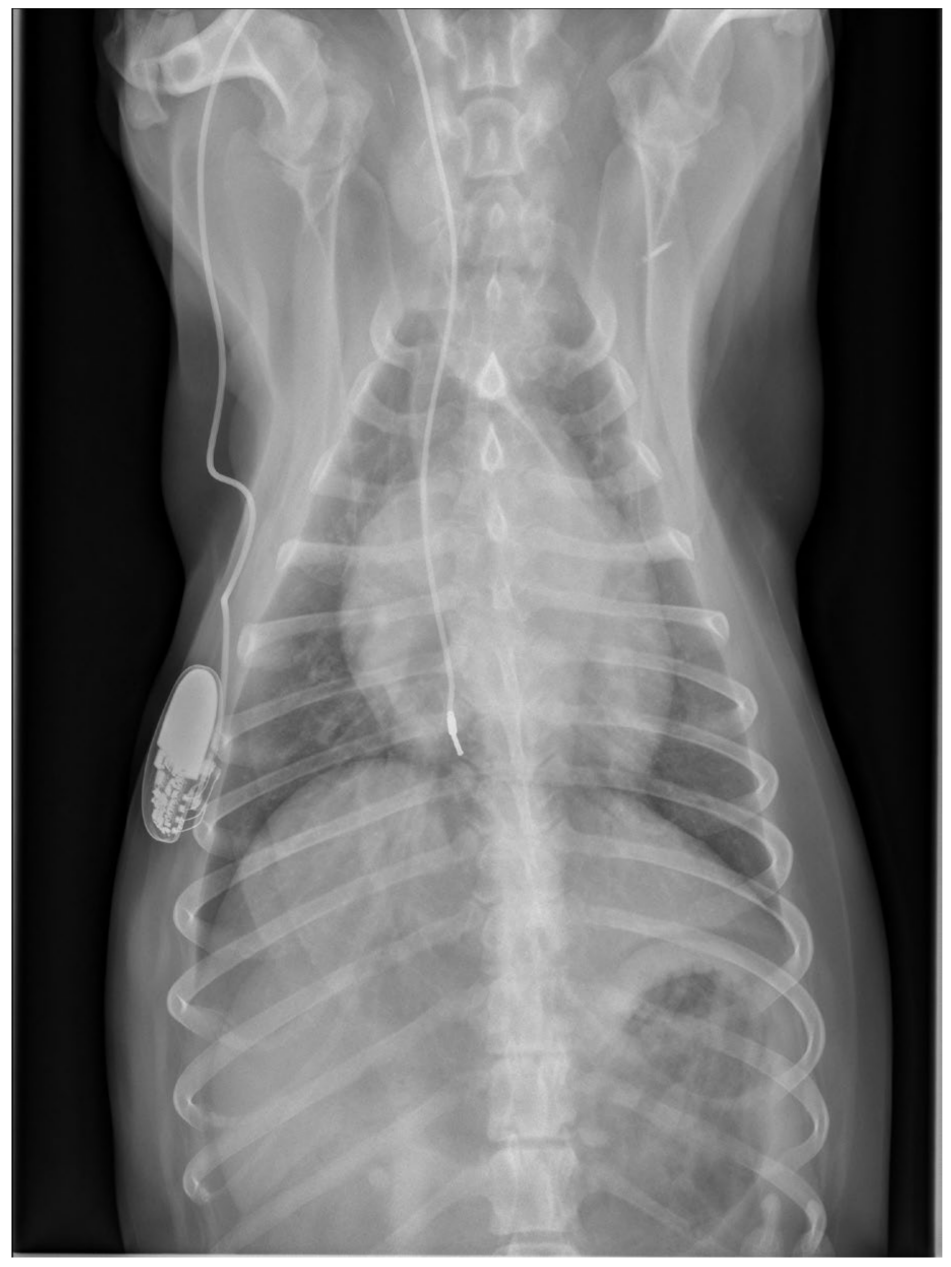
Thoracic radiographs of a dog with chronic cough, hyperthermia and increased respiratory rate show no pulmonary pathology and unchanged position of the pacemaker electrode compared to the direct post-operative radiographs.

**Figure 3:**
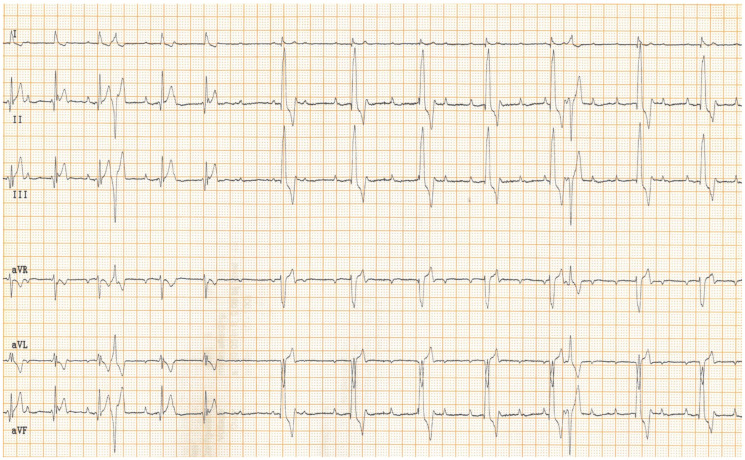
Six-lead surface electrocardiogram of a dog shows two types of basal rhythm. At the beginning of the trace an accelerated idioventricular rhythm is present. In the rest of the tracing, paced QRS complexes are seen with left bundle branch block morphology. The morphology of these latter QRS complexes is identical to the ones shown in Figure 1. Atrio-ventricular dissociation is present in the whole tracing, as is expected with a 3rd-degree atrio-ventricular block and single-chamber pacing with VVI mode. In addition, two solitary ventricular premature complexes are apparent with a right bundle branch block morphology. Paper speed: 25 mm/s, sensitivity: 5 mm/mV.

**Figure 4:**
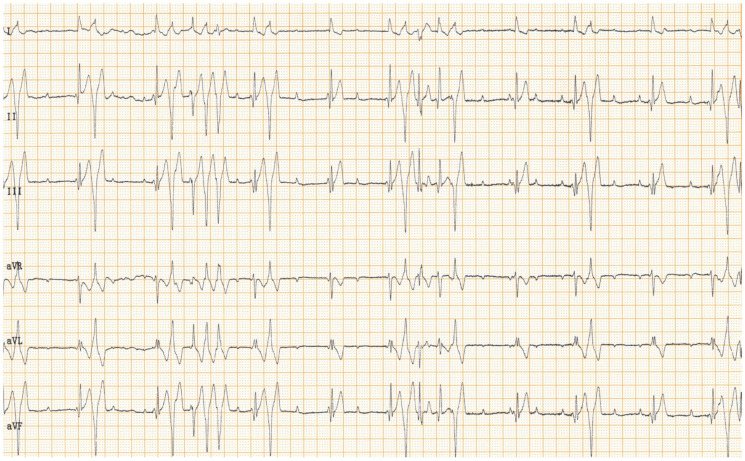
Six-lead surface electrocardiogram of a dog shows an irregular rhythm with atrio-ventricular dissociation, compatible with a 3rd-degree atrio-ventricular block. Instead of paced QRS complexes, this tracing shows an accelerated idioventricular rhythm with short runs of ventricular tachycardia with an instantaneous rate of 300 beats per minute and R-on-T phenomenon. The absence of paced QRS complexes indicates proper sensing of the pacemaker. Paper speed: 25 mm/s, sensitivity: 5 mm/mV.

Physical examination at the authors’ institution, three years after the pacemaker implantation, revealed a bright, alert and responsive dog with a respiratory rate of 60 breaths/min and a pulse rate of 44 beats/min. The rectal temperature was 39.5 °C. The dog had received the last dose of antibiotics a day previously. Thoracic radiographs, an electrocardiogram, an echocardiogram, an abdominal ultrasound examination, urinalysis, and feces and blood tests were performed in order to identify the cause of the cough, hyperthermia and bradycardia. Thoracic radiographs showed no abnormalities and the position of the pacemaker lead was identical to that of the radiographs performed immediately after implantation (Figure 2). Electrocardiography showed a good functioning pacemaker, and ventricular premature complexes with periods of ventricular tachycardia (Figure 3 and Figure 4), which were assumed to have caused the pulse deficit.

**Figure 5:**
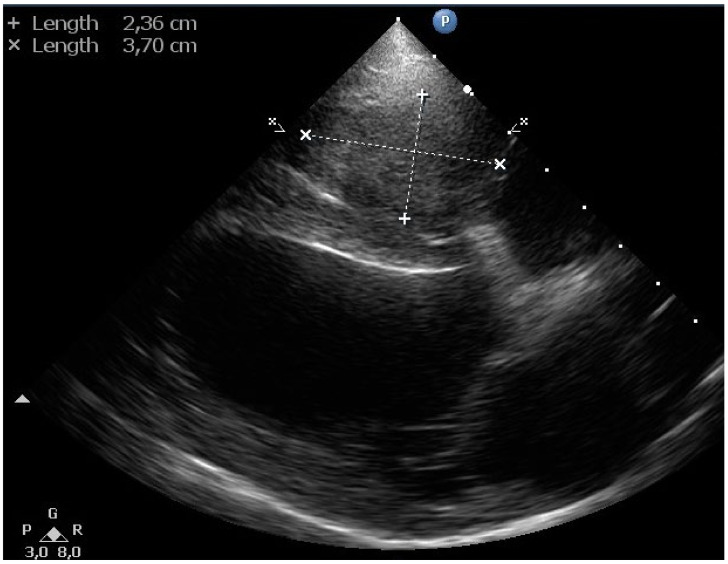
Two-dimensional gray-scale standard right parasternal 4-chamber long-axis echocardiographic image of a dog with a transvenous pacemaker lead shows a soft tissue mass of 2.36 × 3.70 cm (indicated by the calipers), which fills the right ventricular lumen completely.

Echocardiography revealed a soft tissue mass (~2.5 × 3.7 cm) in the right ventricular lumen around the pacemaker electrode (Figure 5). No thrombus in the pulmonary trunk or the pulmonary artery branches was noticed.

Abdominal ultrasonography did not reveal any clinically relevant changes. Feces parasitology (Baermann and flotation) was negative for eggs and larvae of endoparasites. Blood examination showed a mild anemia (hematocrit 0.39 L/L, reference 0.42–0.61 L/L), a severe leukocytosis (45.9 × 10^9^/L, reference 4.5–14.6 × 10^9^/L) due to mature (segmented) neutrophilia (39.5 × 10^9^/L, reference 2.9–11.0 × 10^9^/L). Juvenile neutrophilic granulocyte (bands) count was marginally elevated (0.5 × 10^9^/L, reference 0.0–0.3 × 10^9^/L) and a mild monocytosis was present (2.3 × 10^9^/L, reference 0.0–0.9 × 10^9^/L). Lymphocyte, eosinophilic and basophilic granulocyte counts were within the reference ranges. Total protein concentration was within the reference range (70 g/L, reference: 55–72 g/L) with a mild hypoalbuminemia (20 g/L, reference: 26–37 g/L). After having taken three blood samples for bacterial culture, a course of amoxicillin with clavulanic acid was prescribed for 10 days (375 mg, 15 mg/kg, q12h). Bacterial growth was not seen in any of the three samples for blood culture or in a punctured urine sample. Radiographs of the thoracolumbar vertebrae showed no signs of discospondylitis.

A blood test after one week of antibiotic treatment showed a worsening of the leukocytosis (59.4 × 10^9^/L, reference 4.5–14.6 × 10^9^/L) due to mature (segmented) neutrophilia (49.3 × 10^9^/L, reference 2.9–11.0 × 10^9^/L). Juvenile neutrophilic granulocyte (bands) count was markedly elevated (2.9 × 10^9^/L, reference 0.0–0.3 × 10^9^/L). Monocytosis was still present (3.6 × 10^9^/L, reference 0.0–0.9 × 10^9^/L). Urinalysis of a punctured urine sample showed a good concentrated urine (1035) with a pH of 7.0. Hemoglobin and a moderate amount of protein (4.30 g/L, reference < 0.56 g/L) were found in the urine with a protein/creatinine ratio of 2.02 (reference < 1.00). Microscopic analysis of the urine sediment showed a low number of leukocytes (0–5/field) and erythrocytes (5–15/field).

**Figure 6:**
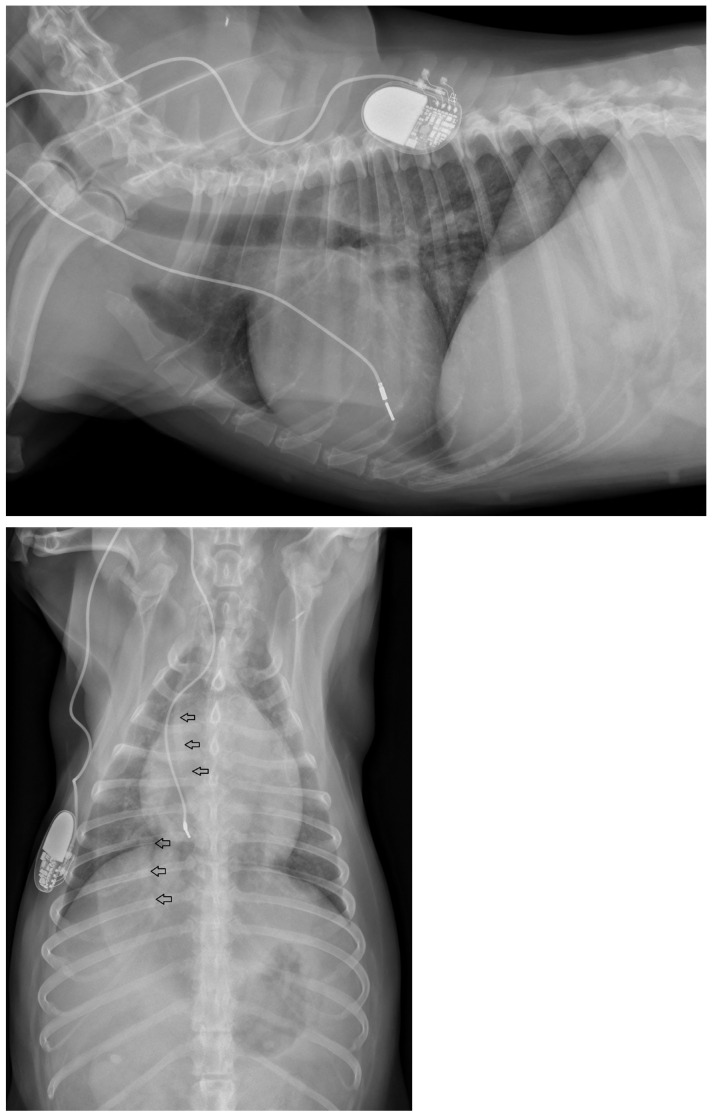
Thoracic radiographs of a dog with chronic cough, hyperthermia and increased respiratory rate and effort show unchanged position of the pacemaker electrode compared to the direct post-operative radiographs. The open arrows indicate the prominent pulmonary artery branches to the right cranial and right caudal lung lobes.

At the next check-up a week later, the owner reported a gradual worsening of appetite and exercise tolerance, while frequent coughing remained unchanged. Every coughing episode was followed by gagging. In addition, the owner reported an increased respiratory rate at rest, frequent panting, and episodes of weakness during walks. Physical examination revealed a slightly depressed dog with an increased respiratory effort and a respiratory rate of 52 breaths/min. The femoral pulses were irregular with a rate of 60 beats/min. Rectal temperature was 38.6 °C. Thoracic radiographs were repeated, which showed a mild diffuse broncho-interstitial lung pattern, sternal lymphadenopathy and increased diameter of the lobar pulmonary arteries to the right cranial and right caudal lung lobes (Figure 6).

A computed tomography scan of the thorax and a diagnostic bronchoscopy were proposed to the owner to further evaluate the cough and tachypnea, and the dog was sent home. The owner returned with the dog the next day to the emergency service because the dog had been coughing and gagging throughout the night, had had a syncopal episode and had developed dyspnea. At presentation, the dog showed signs of respiratory distress; it was weak and had an enlarged abdomen and cyanotic mucous membranes. The owner elected for immediate euthanasia and gave permission for post-mortem examination.

At necropsy, the pulse generator of the pacemaker was located subcutaneously in the right-sided intercostal space at the level of the fifth and sixth ribs. It was surrounded by a connective tissue capsule with no indication of inflammation in the adjacent tissue. The lumen of the right jugular vein was slightly dilated at the site of the entry of the electrode; there was no evidence of thrombus-forming.

**Figure 7:**
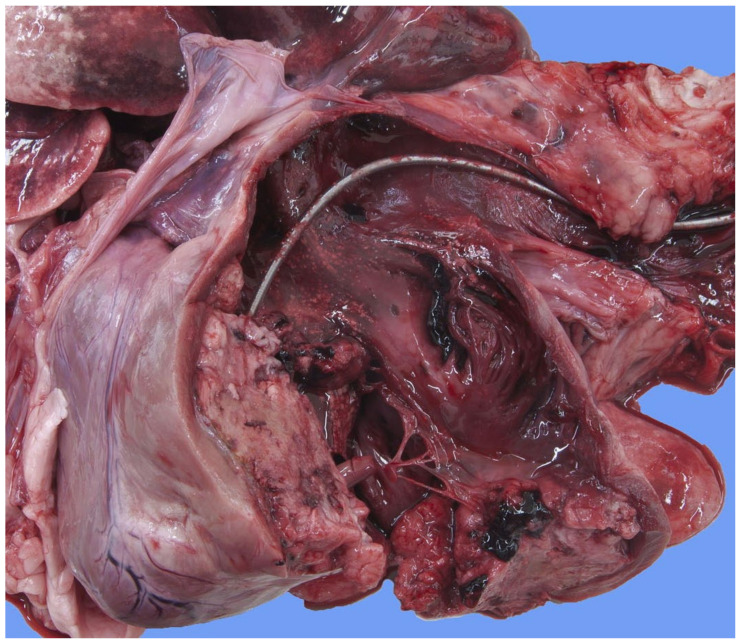
Necropsy shows the right side of the heart and mediastinum of the dog. The walls of the jugular vein, the right atrium and the right ventricle are open allowing the visualization of the lumen. The pacemaker lead disappears in the pale soft tissue mass that fills the right ventricular chamber.

**Figure 8:**
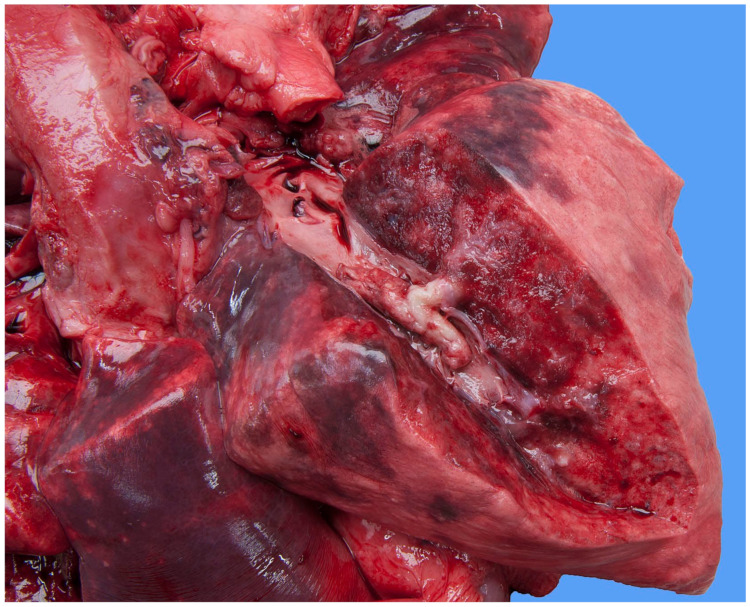
Necropsy shows a large thrombus in the pulmonary artery of the right caudal lung lobe of the dog. The lung parenchyma has an edematous aspect.

Focally extending throughout the right ventricular lumen and partially incorporating the chordae tendineae and tricuspid valve leaflets, was a large (6 × 4 × 4 cm) thrombus adhered to the endocardium. A length of approximately 3 cm of the pacing electrode was embedded within the thrombus (Figure 7). The myocardium of the adjacent interventricular septum and free ventricular wall showed patchy white discoloration. The site of the tip of the electrode was visible in the apex as a small, slightly indented, firm, white area of connective tissue. Thrombus material filled the lumens of the right and left pulmonary arteries (Figure 8). The lungs were mildly congested and edematous. There was moderate congestion in the liver and mild ascites.

**Figure 9:**
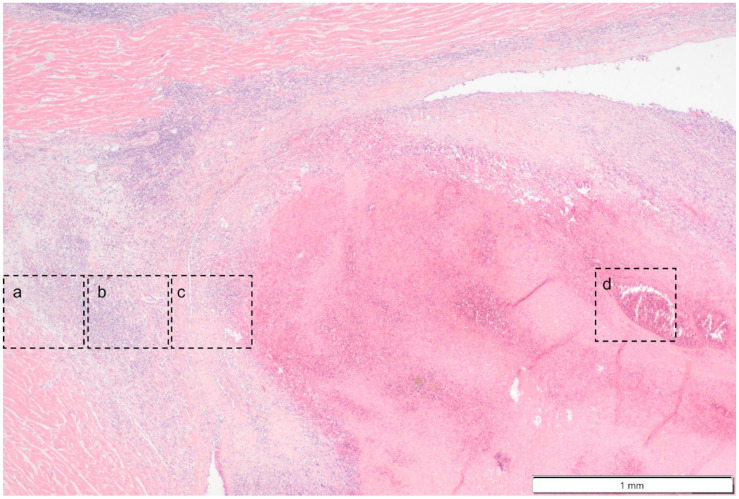
Photomicrograph overview of the attachment site of the chronic thrombus to the endocardium of the right ventricle. Hematoxylin & Eosin staining, 2× magnification. The insets are shown and described in larger magnification in Figure 10.

**Figure 10:**
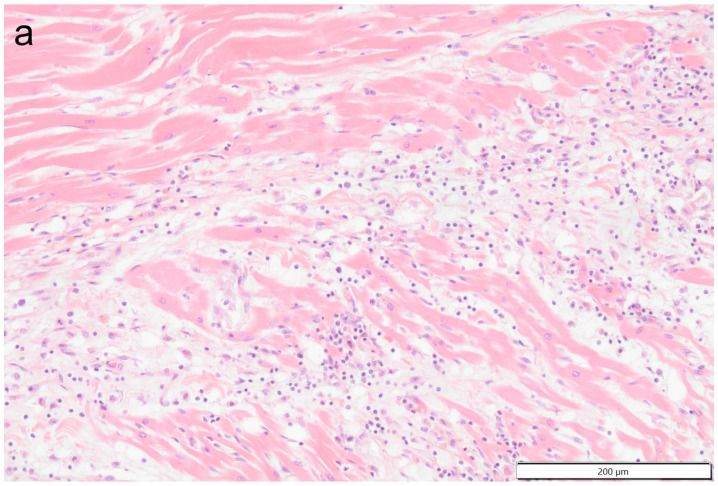

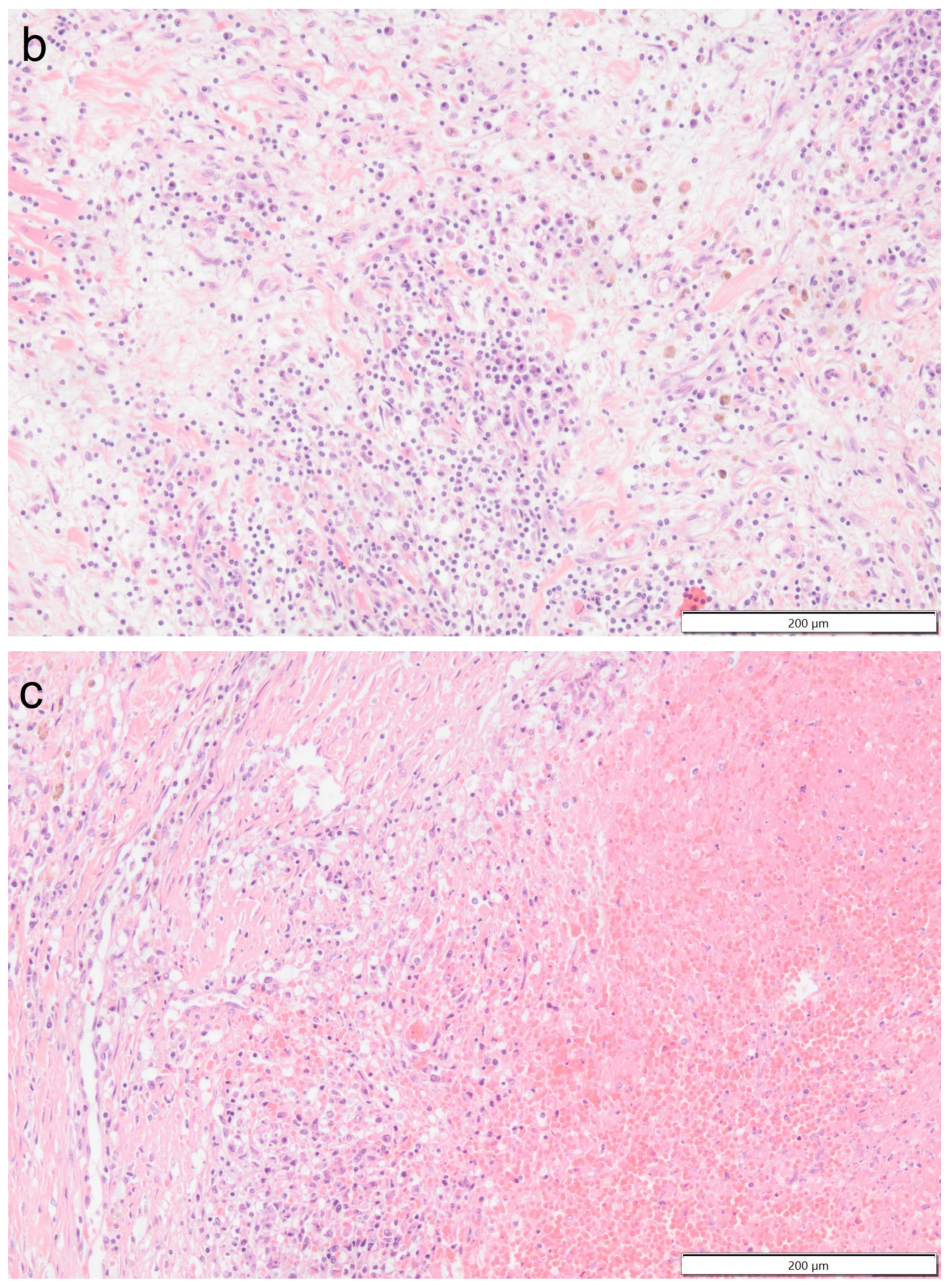

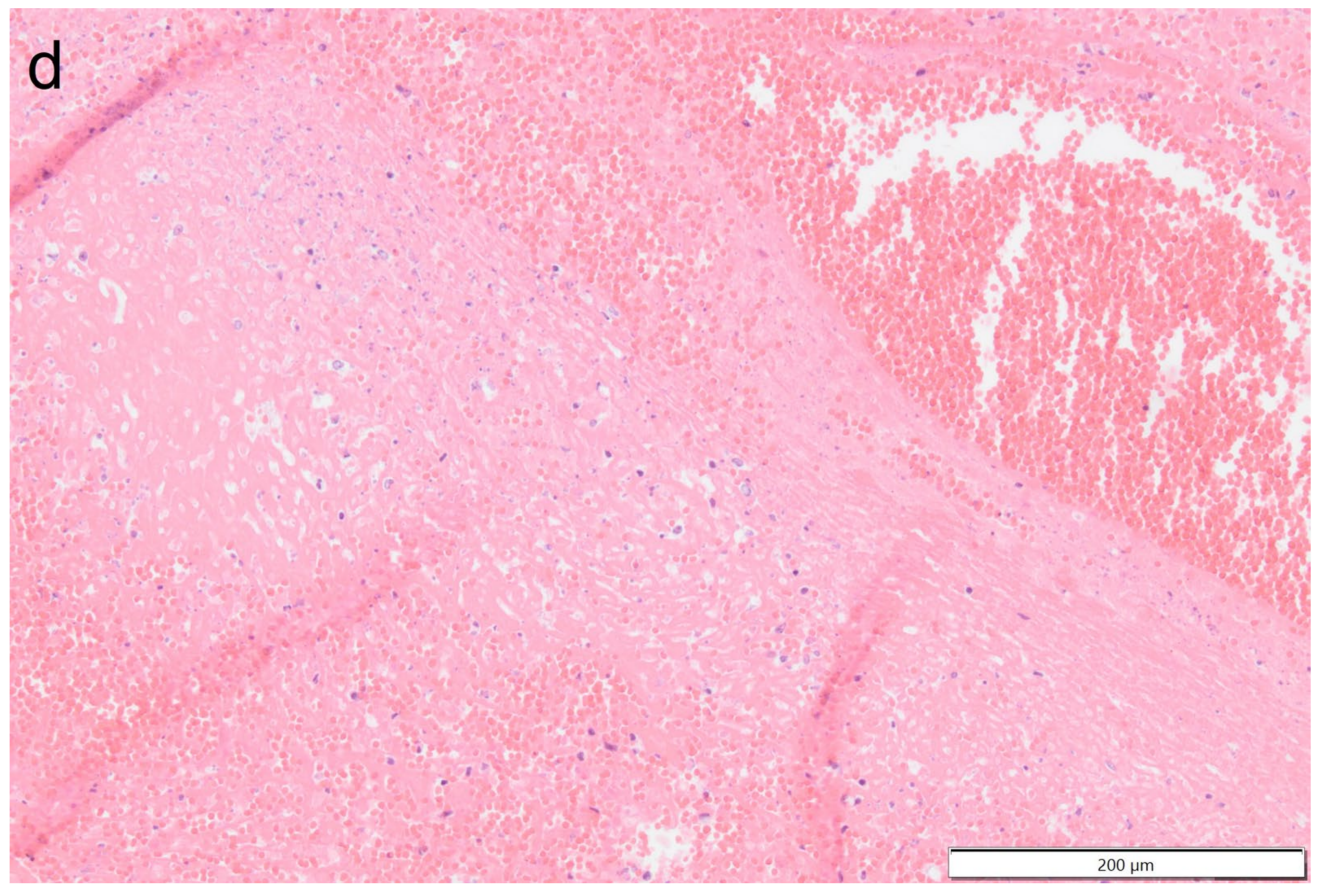
Photomicrographs show the attachment site of the chronic thrombus to the endocardium of the right ventricle. Hematoxylin & Eosin staining, 10× magnification. Marked mononuclear inflammation expands the myocardium (**a**) and (sub)endocardium at the site of thrombus attachment (**b**), which is further expanded by vascularized connective tissue confluent with the periphery of the thrombus (organization, (**c**)). The majority of the thrombus consists of a fibrino-necrotic core with islands of erythrocytes (**d**).

**Figure 11:**
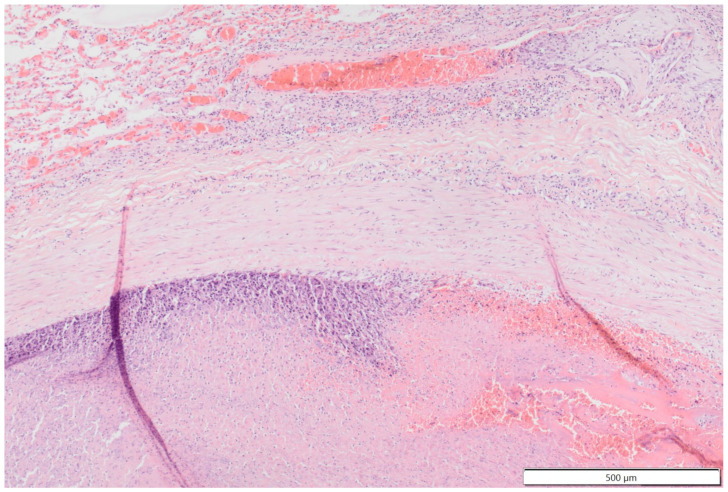

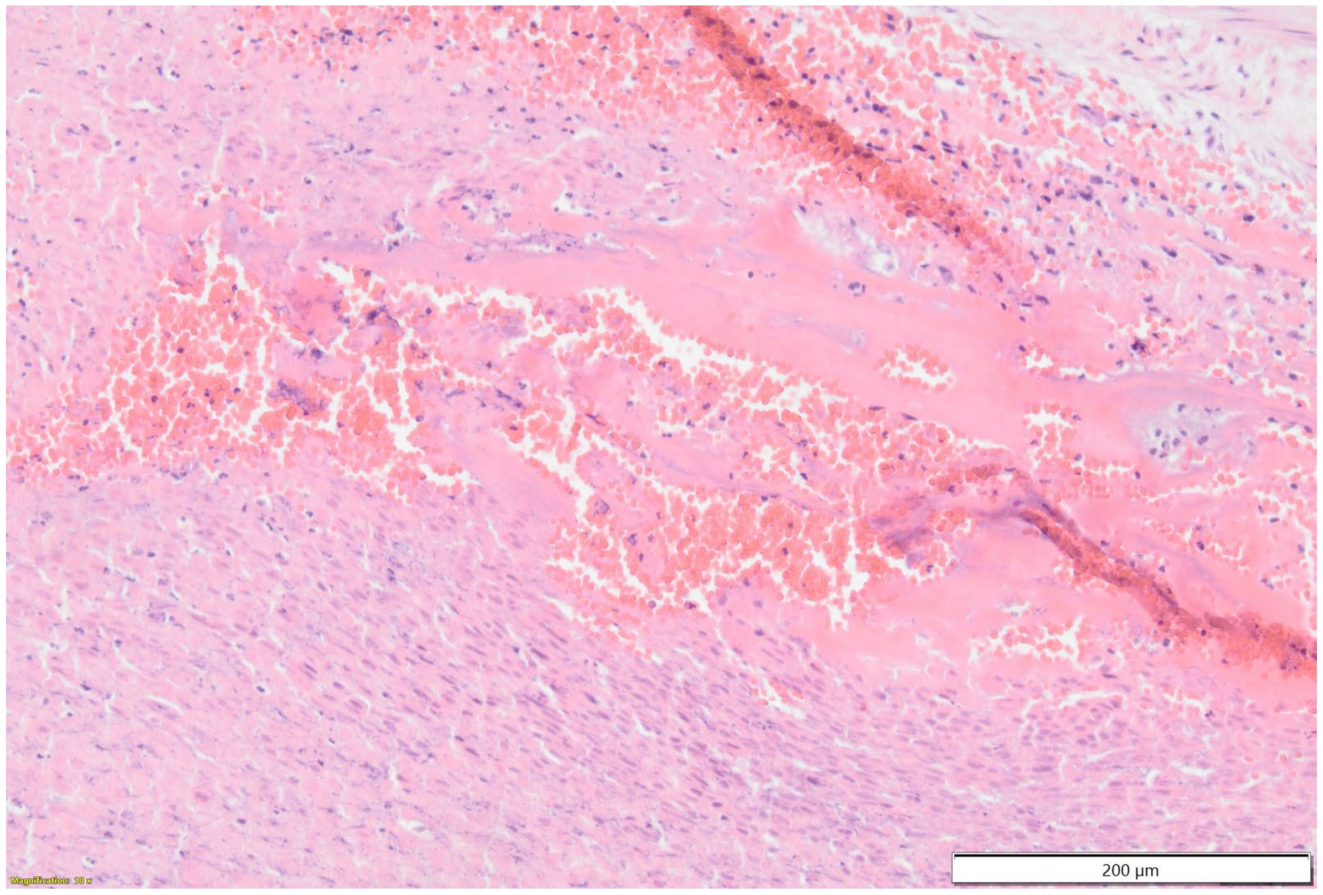
Photomicrographs of the thromboembolus in the pulmonary artery: 2× and 10× magnification images show the periphery with attachment to the endothelium, mild edema of the vascular wall, small-caliber blood-filled channels (recanalization) interspersed between areas of connective tissue, fibrin and inflammatory cells.

**Figure 12:**
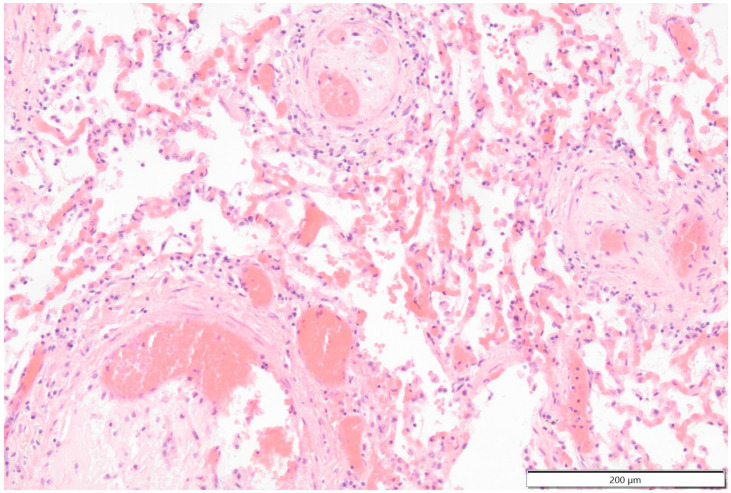
Photomicrograph shows that the lumen of smaller pulmonary arteries is partially occluded by thromboembolic material. Hematoxylin & Eosin staining 40× magnification.

Histologically the right ventricular thrombus was characterized by a massive central zone of amorphous eosinophilic material with occasional scattered cellular debris (fibrin and necrosis) and scattered deposits of hemosiderin and hematoidin pigments. Peripherally, the thrombus consisted of a variably thick layer of connective tissue that was infiltrated by moderate numbers of macrophages, lymphocytes and plasma cells. This inflammatory infiltrate extended into the adjacent myocardium. At the site of electrode insertion, the myocardium was focally replaced by connective tissue and adipose tissue. Multiple coronary vessels had thickened walls that partially occluded the lumens. Multiple pulmonary vessels were obstructed by similar material as described in the right ventricle (thromboemboli) and here this was interspersed with small irregular vascular channels (recanalization) (Figure 9, Figure 10, Figure 11 and Figure 12). Histopathological analysis confirmed marked chronic mural endocarditis and myocarditis with chronic thrombus formation in the right ventricle and pulmonary arteries.

Histologically, the kidneys showed an increased cellularity and mesangial thickening in multiple glomeruli consistent with focal global membranoproliferative glomerulonephritis.

## 3. Discussion

The present case illustrates a severe late-onset complication of permanent transvenous pacemaker implantation in a dog. Pacemaker-lead-associated thrombosis is a well-known complication in humans with an artificial pacemaker [11,12,13,14,15,16,17,18,19,20]. The thrombosis can be present anywhere along the length of the lead when this is located intraluminally, such as in the superior cava vein, the right atrium or the right ventricle. As pacemaker-lead-associated thrombosis often remains clinically silent, the real incidence of this complication in both humans and dogs is unknown. Human studies report it in up to 45% of cases [11,12,16,21], and the recently published multicenter study on dogs reported an incidence of 10% in 260 dogs [10]. In case the pacemaker-lead-associated thrombosis leads to clinical signs, typically due to congestion, treatment can be warranted [21,22,23]. Both anticoagulant and interventional therapies have been reported in dogs [8,9,24].

Protein-losing nephropathy is a known predisposing factor for thrombus formation via losing circulating antithrombin [10,25,26]. Whether the present dog had proteinuria at the pacemaker implantation or it developed later remains unknown, as no urinalysis was performed before pacemaker implantation to screen for protein-losing nephropathy. However, when the dog was presented with clinical signs of a possible systemic infection, urinalysis was performed, and renal proteinuria was detected. Histopathologic examination of the kidneys revealed a membranoproliferative glomerulonephritis post-mortem. If proteinuria had been detected earlier, thrombus formation might have been prevented by daily administration of antithrombotic drugs. Recent guidelines recommend prophylactic antithrombotic treatment for dogs with a transvenous pacemaker and proteinuria [25,26]. Echocardiographic examination during the routine yearly recheck examinations might have detected the right ventricular thrombus formation in an earlier stage in the present case. Because having a pacemaker implanted is not an indication for echocardiography during the rechecks in an asymptomatic dog, this was not performed in the present case either. However, this practice might be changed in future cases based on the findings of the present dog and recent literature [10], especially because with echocardiography a thrombus caused by any hypercoagulable states (such as hyperadrenocorticism or neoplasia) can be detected.

Chronic cough can be a clinical manifestation of many respiratory diseases of the trachea, bronchi and lungs. The etiology in dogs can be infectious (including viral, bacterial, fungal or parasitic causes), non-infectious inflammatory (such as chronic idiopathic bronchitis or eosinophilic bronchopneumopathy), neoplastic (primary or metastatic disease) or structural (such as airway collapse due to tracheobronchomalacia). In the presented dog, the only manifestation of pulmonary thromboembolism was a chronic cough for more than two months. Coughing is also the most common manifestation of heartworm disease in dogs, which has a somewhat similar pathogenesis, through perivascular inflammation, as pulmonary thromboembolism. Because small pulmonary thromboemboli do not necessarily cause radiographic changes, this diagnosis can be easily missed with plain radiographs. Clinical signs of respiratory distress arise only after a massive pulmonary thromboembolism, resulting from ventilation-perfusion mismatch. We speculate that the most likely reason for the cough was local perivascular pulmonary inflammation caused by small pulmonary thromboemboli. In humans, coughing is a reported manifestation of pulmonary thromboembolism; however, the etiology is unknown [27]. The exercise intolerance, episodic weakness and dyspnea in the presented dog were most likely the result of systemic hypoxemia and assumed systemic arterial hypotension secondary to presumed pulmonary arterial hypertension due to the massive pulmonary thromboembolism.

In the present dog, a number of clinical findings were compatible with an inflammatory, potentially infectious, process, such as hyperthermia, severe peripheral leukocytosis with left shift and the development of runs of ventricular tachycardia, the latter suggesting myocardial damage. However, hyperthermia and severe leukocytosis can also result from non-infectious inflammatory and neoplastic causes. In our case, the negative blood cultures and the lack of clinical response to various antibiotic courses made a bacterial etiology less likely. However, an underlying bacterial infection cannot be completely excluded because the negative blood culture could have been due to prior antibiotic use. In this case, the lack of clinical response to antibiotic therapy could have been related to the severity of pulmonary thromboembolism.

The development of malignant ventricular tachycardia was a highly suggestive ante-mortem sign of myocarditis [2]. Together with the soft-tissue mass in the right ventricle, this finding was suggestive of an infectious complication, such as a bacterial mural endo- and myocarditis, and pacing lead infection. The histologically determined chronicity of both the thrombus and the myocardial inflammation means that it is not possible to say with certainty which was the initiating pathology. It is possible that a viral myocarditis led to endothelial damage and intracardiac thrombosis [28,29]; similarly possible and unproven is that the myocardial inflammation extended from the site of the thrombus attachment as a part of the thrombus organizing process. The latter scenario seems more likely, as viral myocarditis would have been apparent in the whole heart and not only adjacent to the thrombus. As part of a thrombus organization process, a large number of leukocytes accumulate in the adjacent vital tissue, which, in this case, was the right ventricular wall. Consequently, myocarditis develops, which can give rise to ectopic beats, in the present case, premature ventricular complexes and even runs of ventricular tachycardia.

Due to the rapid clinical deterioration of the dog in this case report, no ante-mortem diagnosis was reached. Therefore, no anticoagulant therapy was initiated. In an earlier disease stage, rivaroxaban could have been started (1–2 mg/kg/day PO) until serial (bi-monthly) echocardiographic examinations revealed the resolution of the thrombus. Simultaneously, but life-long administration of clopidogrel (1–3 mg/kg q24h) could have been considered as a prophylactic antiplatelet agent [30].

## 4. Conclusions

Coughing in a dog, especially with an implanted transvenous pacemaker, could be a clinical sign of pulmonary thromboembolism. When pacemaker-lead-associated thrombosis is suspected with echocardiographic examination, a blood culture should be performed to rule out bacterial infection of the pacing lead. With these test results, prolonged courses of antibiotic treatment can be prevented. Runs of ventricular tachycardia can develop as a result of intracavitary thrombus without the presence of an infectious mural endocarditis or myocarditis. Secondary endo- and myocarditis might result from the organization process of an intracardiac thrombus. Screening for proteinuria before endocardial pacemaker implantation in dogs may help to minimize the risk of lead-associated thrombosis by the initiation of life-long daily oral prophylactic antiplatelet therapy in case of a confirmed severe renal proteinuria. Screening for proteinuria should also be considered in dogs with implanted endocardial pacing leads at the yearly routine serial pacemaker rechecks.

## Data Availability

Data are available from the corresponding author upon reasonable request.
